# STAT3 Modulation to Enhance Motor Neuron Differentiation in Human Neural Stem Cells

**DOI:** 10.1371/journal.pone.0100405

**Published:** 2014-06-19

**Authors:** Rajalaxmi Natarajan, Vinamrata Singal, Richard Benes, Junling Gao, Hoi Chan, Haijun Chen, Yongjia Yu, Jia Zhou, Ping Wu

**Affiliations:** 1 Department of Neuroscience and Cell Biology, The University of Texas Medical Branch, Galveston, Texas, United States of America; 2 Department of Pharmacology & Toxicology, The University of Texas Medical Branch, Galveston, Texas, United States of America; 3 Department of Radiation Oncology, The University of Texas Medical Branch, Galveston, Texas, United States of America; Temple University School of Medicine, United States of America

## Abstract

Spinal cord injury or amyotrophic lateral sclerosis damages spinal motor neurons and forms a glial scar, which prevents neural regeneration. Signal transducer and activator of transcription 3 (STAT3) plays a critical role in astrogliogenesis and scar formation, and thus a fine modulation of STAT3 signaling may help to control the excessive gliogenic environment and enhance neural repair. The objective of this study was to determine the effect of STAT3 inhibition on human neural stem cells (hNSCs). *In vitro* hNSCs primed with fibroblast growth factor 2 (FGF2) exhibited a lower level of phosphorylated STAT3 than cells primed by epidermal growth factor (EGF), which correlated with a higher number of motor neurons differentiated from FGF2-primed hNSCs. Treatment with STAT3 inhibitors, Stattic and Niclosamide, enhanced motor neuron differentiation only in FGF2-primed hNSCs, as shown by increased homeobox gene Hb9 mRNA levels as well as HB9^+^ and microtubule-associated protein 2 (MAP2)^+^ co-labeled cells. The increased motor neuron differentiation was accompanied by a decrease in the number of glial fibrillary acidic protein (GFAP)-positive astrocytes. Interestingly, Stattic and Niclosamide did not affect the level of STAT3 phosphorylation; rather, they perturbed the nuclear translocation of phosphorylated STAT3. In summary, we demonstrate that FGF2 is required for motor neuron differentiation from hNSCs and that inhibition of STAT3 further increases motor neuron differentiation at the expense of astrogliogenesis. Our study thus suggests a potential benefit of targeting the STAT3 pathway for neurotrauma or neurodegenerative diseases.

## Introduction

Acute spinal cord injury (SCI) and amyotrophic lateral sclerosis (ALS) are characterized by death of cholinergic motor neurons accompanied by reactive astrogliosis, *i.e.* hypertrophy and proliferation of astrocytes and alterations in their gene expression patterns. Typically, after spinal cord injury, initial motor neuron death is mediated by mechanical or physical forces. The massive death of residual neurons is due to secondary apoptotic, necrotic and excitotoxic processes, which initiate cascades of neuro-inflammatory responses by proinflammatory molecules, leading to reactivation and proliferation of nearby astrocytes. Similarly, prominent astrogliosis is a pathological hallmark of ALS in humans and animal models. For instance, transgenic rats carrying SOD1^G93A^ mutation exhibited astrogliosis along with the loss of ventral motor neurons and astrocytic glutamate transporter [1], [2]. Moreover, recent studies show that astrocytes derived from familial and sporadic ALS patients exhibit non-autonomous toxicity to motor neurons [3], [4]. Thus, it is clear that increased astrogliosis resulting from acute spinal injury or chronic neurodegenerative conditions creates a highly gliogenic cellular environment, which is not conducive to the formation or long-term survival of motor neurons. Hence, in such patients, potential therapy should employ a two-pronged approach: 1) reduce the local gliogenic environment and 2) switch the environmental milieu such that it promotes/sustains neurogenesis.

In rodent models of SCI, levels of pro-inflammatory interleukin such as IL-6 peak acutely in the injured areas and lead to activation of the JAK1-STAT3 signaling pathway, which contributes to development of neuropathic pain [5], [6]. Moreover, in previous work, conditional ablation of STAT3 increased motor deficits after spinal cord injury [7]. STAT3 signaling is also upregulated in certain neurodegenerative diseases. For instance, spinal cord microglia, reactive astrocytes and motor neuron nuclei of ALS patients showed increased levels of phosphorylated STAT3 [8]. ALS mouse models also exhibited persistent activation and nuclear translocation of phosphorylated STAT3 [9]. Together these studies support the hypothesis that after SCI, and perhaps in neurodegenerative conditions, activation of STAT3 signaling causes various undesirable outcomes. Hence, to promote neurogenesis in these tissues, it might be important to inhibit STAT3 activity. However, this hypothesis must be considered in the light of a growing body of literature suggesting that STAT3 is an injury-induced signaling mechanism critical for various aspects of nerve regeneration [7], [10]–[13]. For instance, intrathecal administration of STAT3 inhibitors after nerve injury or spinal ligation reduced symptoms of neuropathic pain in rats [6], [10]. Moreover, it is known that *in vitro* suppression of STAT3 [11] or its conditional deletion *in vivo* [12] induces neurogenesis and inhibits astrogliosis. Thus, it appears that the key to enhance neurogenesis and thereby, neural repair in SCI or ALS patients, would be to precisely regulate STAT3 pathway, such that it is inhibited at exactly the right time and to the right levels.

For stem cell researchers, replacing lost motor neurons in SCI or ALS patients by either transplantation of exogenous neural stem/progenitor cells or modulating endogenous adult stem cells to produce new motor neurons *in vivo* is a desirable but challenging goal. Previous studies from our laboratory have shown that the fate of human neural stem cells (hNSCs) can be modulated by precise amounts of certain growth factors in the surrounding environment [14], [15]. For instance, human fetal brain-derived NSCs primed with basic fibroblast growth factor (FGF2), heparin and laminin (FHL) differentiated into cholinergic motor neurons [14], [15], whereas epidermal growth factor (EGF), leukemia inhibitory factor (LIF) and laminin (ELL)-primed hNSCs generated glutamate and γ-aminobutyric acid (GABA) neuronal subtypes and showed minimal capability to form cholinergic motor neurons [15].

Thus, spinal damage from injury or degenerative disease causes loss of motor neurons, astrogliosis and a significant increase in STAT3, which increases astrocyte activation. In this study, we asked if pharmacological inhibition of STAT3 in the presence of FGF2 will enhance differentiation of hNSCs to motor neurons and reduce astrocytosis in an *in* *vitro* paradigm, more amenable to molecular analyses. Among the commercially available STAT3 inhibitors, we chose Stattic and Niclosamide because of their potency and relative specificity in inhibiting the STAT3 signaling pathway. Stattic has been shown to specifically inhibit STAT3 over STAT1, STAT5, c-Myc/Max, Jun/Jun and Lck in HepG2 cell lines [16]. Similarly, Niclosamide displays selectivity for STAT3 over STAT1, STAT5, JAK1, JAK2 and Src kinases [17]. Thus far, no studies have reported the effectiveness of these inhibitors in obtaining neurons from neural stem cells. This study shows that blocking STAT3 activity in FGF2-primed hNSCs increases expression of markers specific to motor neurons and decreases astrocyte markers, presumably by blocking nuclear transport of tyrosine-phosphorylated form of STAT3.

## Methods/Materials

### Human NSC Culture

Human fetal brain NSCs, the line K048 generously provided by C.N. Svendsen as we previously described [14], were cultured as free-floating “neurospheres” in 75-cm^2^ flasks with growth medium containing DMEM (high glucose, L-glutamine)/Ham’s F12 (3∶1; Invitrogen/Gibco, Grand Island, NY), and supplemented with 15 mM HEPES (Sigma, St. Louis, MO), 1.5% D-glucose (Sigma), 67 IU/ml/67 µg/ml penicillin/streptomycin (Mediatech, Inc/CellGro, Manassas, VA), 25 µg/ml bovine insulin (Sigma), 100 µg/ml human transferrin (Sigma), 100 µM putrescine (Sigma), 20 nM progesterone (Sigma), 30 nM sodium selenite (Sigma), 20 ng/ml recombinant human EGF (Invitrogen), 20 ng/ml recombinant human FGF2 (R&D Systems, Minneapolis, MN), 5 µg/ml heparin (R&D Systems), 10 ng/ml recombinant human LIF (Millipore), and 2 mM L-glutamine (Sigma). Cells were passaged every 7 days. Cultured spheres were pelleted by centrifugation at 216×g for 10 min, resuspended in 0.025% trypsin (Sigma) plus 1.5% D-glucose dissolved in calcium- and magnesium-free Dulbecco’s phosphate buffered saline (CMF-dPBS, CellGro), and incubated for 10 min at 37°C with periodic trituration. 250 U/ml DNAse was added to break down aggregated DNA in cases of excessive cell lysis. The reaction was stopped by using 1.2 mg/ml of trypsin inhibitor (Sigma) diluted in conditioned medium that was spared from the original cell culture. Cells were mechanically triturated with a 5-ml serological pipet till no visible clumps. Cells were quantified using trypan blue exclusion and hemacytometer counting. After passaging, 5×10^6^ cells were seeded into a 75-cm^2^ flask in 5 ml of conditioned and 10 ml of fresh medium and incubated at 37°C with 8.5% CO_2_ to maintain pH 7.2–7.5. Prior to plating cells, new flasks were treated with conditioned medium (7 ml/75 cm^2^) at 37°C for at least 1 hr, which helped to prevent initial adhesion of cells to the bottom of new culture flasks.

### Priming, Inhibitor Treatment and Differentiation

Priming of hNSC was carried out in 25 cm^2^ flasks (T25 flasks). These flasks were precoated with 2 mL of 0.1 mg/mL poly-D-lysine (Sigma) for one hour and then with 2 mL dPBS containing 1 µg/cm^2^ laminin overnight. Approximately 2 million cells were plated per T25 flask, and primed with either FGF2/heparin/laminin (FHL) or EGF/LIF/laminin (ELL) medium, based on our previous experiments revealing the effects of different priming media on motor neuron differentiation [15]. The priming was carried out for either 1 day for Western blot analyses of protein phosphorylation or 4 days for mRNA measurement by RT-PCR and motor neuron differentiation by immunostaining (see below).

Specific inhibitors for STAT3, Stattic, Niclosamide or HJC0142, were applied to hNSCs during the priming period. After screening various compounds, we decided to use Stattic (Tocris Biosystems, Elsville, MO), Niclosamide (Tocris Biosystems) and HJC0142 (developed in house [18], [19]). We tested Stattic with the following dosages for hNSC treatments: 0.5 µM, 2 µM after researching various dosages and utilizing the IC_50_. We tested Niclosamide with the following dosages: 0.25 µM, 0.5 µM, 1 µM. Stattic was added every day during the 4-day priming period, whereas Niclosamide was administered once. After testing various concentrations, 0.5 µM HJC0149 was selected for further experiments.

### Semiquantitative Reverse Transcription-Polymerase Chain Reaction (RT-PCR)

Cells were washed with PBS, and total RNA was extracted using Qiagen RNA Isolation Kit (Qiagen, Valencia, CA). Reverse transcription was performed using the High Capacity cDNA Reverse Transcription Kit (AB Applied Biosystems, Carlsbad, CA) according to the manufacturer’s instructions. Twenty ng of cDNA was used in the following PCR reaction using empirically determined conditions to allow a quantitative analysis [15]. PCR amplification was performing using REDTaq DNA Polymerase (Sigma) with a program of 95°C for 5 minutes, 35 cycles of 94°C for 30 seconds, 65°C for 1 minute, 72°C for 1 minute, an extension of 72°C for 5 minutes for Hb9 amplification; a program of 95°C for 5 minutes, 23 cycles of 94°C for 30 seconds, 60°C for 30 seconds, 72°C for 90 seconds, and an extension of 72°C for 5 minutes was used for GAPDH amplification. The following reverse and forward primers were utilized: HB9: AGCTGGGCCGGCACCTTCC and CCGCCGCCGCCCTTCTGTTTCTC; GAPDH: TGAAGGTCGGAGTCAACGGA and GATGGCATGGACTGTGGTCAT. PCR products were visualized on an ethidium bromide-stained1.5% agarose gel and the bend intensities under UV light were captured and analyzed with a Chemi-Imager 4400 v5.5 with the Alpha-Ease software. The HB9 expression levels were normalized to that of GAPDH.

### Western Blot Analysis

T25 flasks pre-coated with 0.01% poly-D-lysine/dPBS and 0.5 µg/cm^2^ Laminin/dPBS for 1 hr at 37°C respectively were plated with ∼2.2 million cells. The cells were primed with FHL or ELL along with various concentrations of STAT3 inhibitors, Stattic (0.5 µM, 2 µM) and Niclosamide (0.25 µM and 2 µM) for 24 hrs. The cells were washed with 4 ml dPBS and incubated with 150 µl of solution containing 1x cell lysis buffer (Cell Signaling Technology, Danvers, MA) and 1 mM PMSF (Sigma). The cells were scraped and the lysate rocked for 1 hr at 4°C. Subsequently, the lysate was centrifuged at 14,000×g at 4°C for 10 min and the supernatant was collected in a fresh microfuge tube. Lysates were either used fresh or stored at −80°C. Protein concentration was determined using Bio-Rad Protein Assay. Samples were warmed in a solution containing LDS sample buffer and NuPAGE reducing buffer at 75°C and 2 µg of protein was loaded per well. Proteins were separated using NuPAGE 4–12% bis-tris gel (Invitrogen) electrophoresis (110 V for 90 min) using the recommended running buffer. The proteins were transferred onto a nitrocellulose membrane by running at 120 V/2 h. The blots were blocked with 5% phosphoBLOCKER (for phosphoproteins) or with non-fat dry milk (for regular proteins) for 1 hr at room temperature. The blots were incubated overnight with the following primary antibodies (Cell Signaling Technology): rabbit anti-pSTAT Tyr-705 (1∶1,000), rabbit anti-pSTAT3 Ser 727 (1∶1,000), total rabbit anti-STAT3 (1∶1,000) and mouse anti-GAPDH (1∶1,000). The membranes were washed 3×10 min in TBST with vigorous shaking, followed by incubating with HRP-conjugated secondary antibodies: goat anti-rabbit (1∶5,000) and sheep anti-mouse (1∶2,000) for 1 hr at room temperature. The membranes were rinsed again as before, and incubated with ECL or ECL Plus reagents (for phosphoproteins, Amersham). The bend intensities were measured and analyzed with an Alpha Innotech Imager.

### Immunofluorescence

Cells were primed on glass coverslips in 24-well plates (precoated as described in the priming section) with 0.12 million cells per well. We tested two dosages along with controls: 0.25 µm Niclosamide and 0.5 µm Stattic. Cells stayed in either FHL or ELL medium for 4 days (Stattic treatment was added every day at the same time) and were placed in 1X B27 medium for differentiation for 9 days. B27 medium was made from 50x B27 (Gibco/Invitrogen) and dPBS. After 9 days, cells were fixed in ice-cold 4% paraformaldehyde for 20 min and washed with PBS, and post-fixed with methanol, and then washed with TBS 3 times for 5 minutes each. Cells were permeabilized using 0.25% Triton-X and blocked using 5% Normal Goat Serum. Slides were incubated overnight with the primary antibodies against MAP2 (Chemicon, 1∶500) and Hb9 (Santa Cruz Biotechnology, Santa Cruz, CA, 1∶100) in a double labeling, GFAP (Chemicon, 1∶1,000), pSTAT3-Tyr 705 (Cell signaling Technology, 1∶100) or pSTAT3-Ser727 (Cell signaling Technology, 1∶100). After three washes with TBS for 15 minutes each, cells were incubated in the following secondary antibodies depending on the type of primary antibody: Alexa 568 goat anti-mouse antibody (Invitrogen, 1∶400) or Alexa 488 goat anti-rabbit antibody (Invitrogen, 1∶400). Cells were counter-stained with DAPI to locate the nucleus, and then coverslips were mounted on microscope slides using Fluromount-G. Cells were visualized by using fluorescence or confocal microscope. At least 10 randomly-selected fields of cells were imaged per treatment condition for every experiment and each experiment was independently performed 3–4 times. Images for each fluorophore are achieved individually. The total cell number in each field is determined by DAPI staining. Cells with clear nuclear Hb9 staining were counted as Hb9^+^. Cells that exhibited distinct neurite extensions with MAP2 labeling were counted as MAP2^+^. The ratio of Hb9^+^/MAP2^+^ is calculated accordingly. The localization of active STAT3 is examined with anti-pSTAT3-Y705 antibody. The pY705 immunoreactivity was quantified by an NIS-Element software to determine the average ratio of pY705 immunoreactivity inside a DAPI-labeled nucleus over pY705 in the whole cell.

### WST-1 Assay

Cells were primed and then incubated in 37°C at 8.5% CO_2_ for 72 hours (approximately 0.03 million cells/well) in a 96-well plate in either FHL or ELL medium. After 72 hrs, cells were retrieved and approximately 10 µl of WST-1 reagent (Roche Applied Science, Indianapolis, IN) was added to each well. After approximately 2–3 hours (or after noticing a considerable color change) the plate was moved to the microplate reader, which provided an output spectrophotometric analysis at a wavelength of 450 nm.

### Statistical/Data Analysis

All experiments were repeated at least three times. Results were analyzed with Prism software (GraphPad Software, San Diego, CA). Data are shown as mean±S.E.M and are compared with One-way ANOVA followed by post-hoc tests, or Student’s *t*-test. A *p* value <0.05 is considered statistically significant.

## Results

### FGF2 Priming Reduces Tyrosine Phosphorylated STAT3 in hNSCs

**Figure 1:**
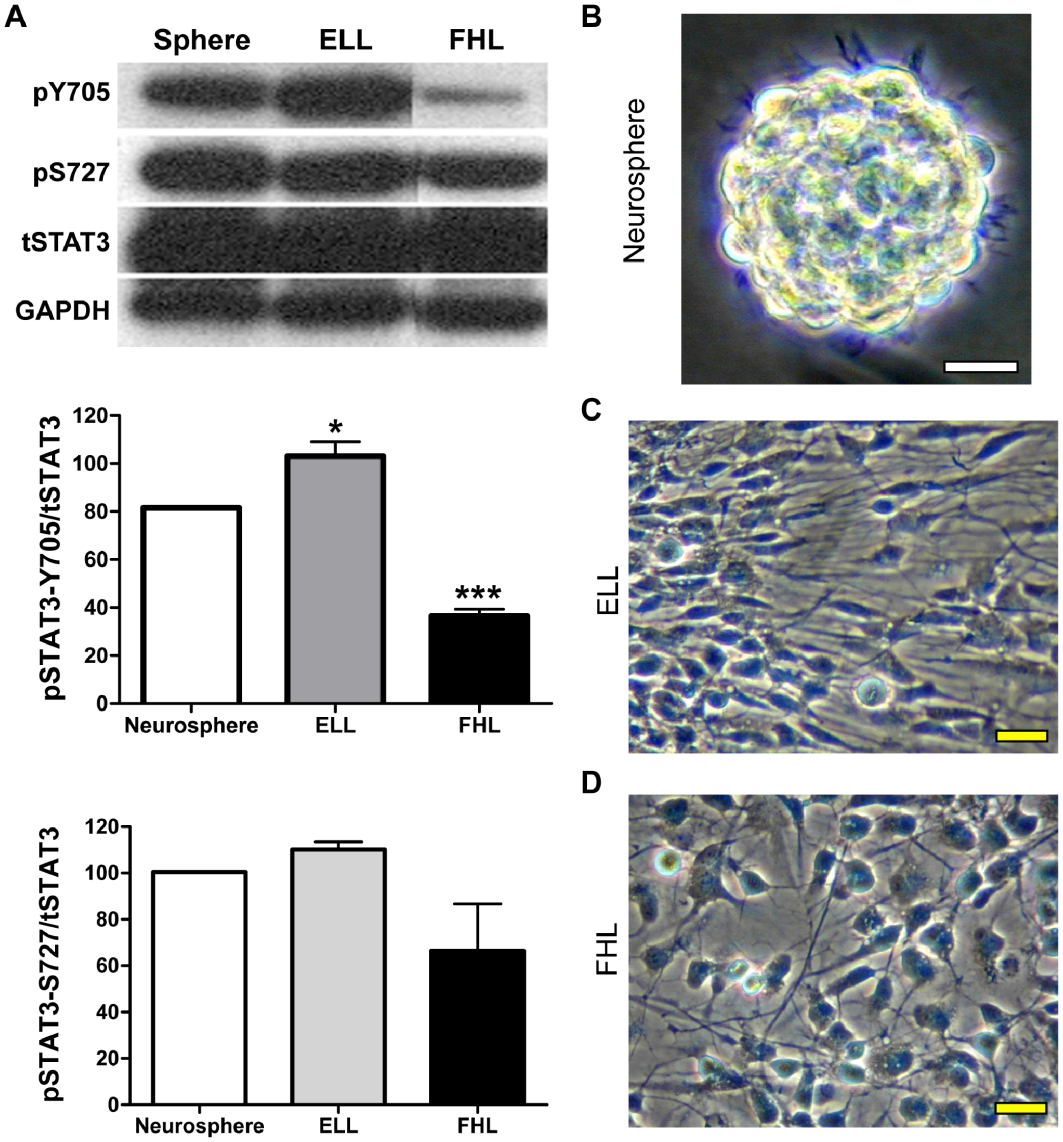
EGF and FGF2 have opposite effects on STAT3 phosphorylation in hNSCs. (**A**) Lysates from neurospheres as well as hNSCs primed with either EGF (ELL) or FGF2 (FHL) for 24 hrs were analyzed by immunoblotting using antibodies against phosphorylated STAT3-Tyr705 (pY705) or STAT3-Ser727 (pS727). The blots were stripped and re-probed for total STAT3 (tSTAT3) and GAPDH (loading control). Densitometric analysis was performed and ratios of phosphorylated STAT3 to total STAT3 (pSTAT3/tSTAT3) are graphically represented as Mean ± SEM (n = 3). *p<0.05 and ***p<0.001 by One-way ANOVA with Tukey post-hoc test. pSTAT3-Y705 is increased in EGF-primed cells, but reduced in FGF2-primed cells when compared to unprimed neurospheres. pSTAT3-S727 levels, however, remain unchanged after either priming. Representative phase contrast images of primary culture hNSCs are shown as neurospheres (**B**), one day after ELL- (**C**) or FHL-priming (**D**). Scale bars, 20 µm. hNSCs: human neural stem cells; ELL: EGF plus LIF and laminin; FHL: FGF2 plus heparin and laminin.

Our previous studies showed that growth factors, EGF and FGF2, have opposite effects on specification of motor neurons from hNSCs, via differential actions on the PI3K/Akt/GSK3B pathway [20]. To determine if priming of hNSCs with FGF2 or EGF affects the activation of STAT3, we performed Western blot analysis using phospho-specific antibodies: tyrosine 705 (pSTAT3-Y705) or serine 727 (pSTAT3-S727). Lysates from neurospheres and hNSCs primed with either FGF2/Heparin/Laminin (FHL) or EGF/LIF/Laminin (ELL) for 24 hr were probed with antibodies against pSTAT3-Y705 or pSTAT3-Ser 727 (Fig. 1A). Densitometric analysis was performed to measure pSTAT3-Y705 or pSTAT3-S727 levels and the ratios of pSTAT3-Y705/total STAT3 or pSTAT3-S727/total STAT3 were graphically represented (Fig. 1A). We found that unprimed hNSCs grown as free-floating neurospheres (Fig. 1B) had considerable amounts of pSTAT3-Y705, pSTAT3-S727 and total STAT3 (Fig. 1A, sphere). As expected, a further 20% increase in the amount of pSTAT3-Y705 was observed upon EGF priming (Fig. 1A, ELL) while there was no change in the amounts of pSTAT3-S727 levels (Fig. 1A, ELL). Upon ELL priming, the cells attained the expected long, radiating morphology with limited migrating potential (Fig. 1C). On the other hand, priming with FGF2 (in the absence of EGF) consistently yielded a 50% reduction in the levels of pSTAT3-Y705 compared to unprimed neurospheres (Fig. 1A, FHL). This effect of FGF2 was specific to tyrosine phosphorylation because pSTAT3-S727 levels remained unchanged upon FGF2 priming (Fig. 1A, FHL). Moreover, as expected, FGF2-treated cells exhibited uniform sizes with more pyramidal morphology (Fig. 1D). Thus, this indicates that stimulation with FGF2 specifically decreases tyrosine 705-, but not serine 727-, phosphorylation of STAT3. Moreover, it also shows that stimulation of hNSCs with EGF results in a moderate upregulation of tyrosine 705-, but not serine 727-, phosphorylation of STAT3. Thus, it appears that in hNSCs only tyrosine phosphorylation of STAT3 is responsive to growth factors.

### STAT3 Inhibitors, Stattic and Niclosamide, Exhibit Dose-dependent Viability in Human Neural Stem Cells

**Figure 2:**
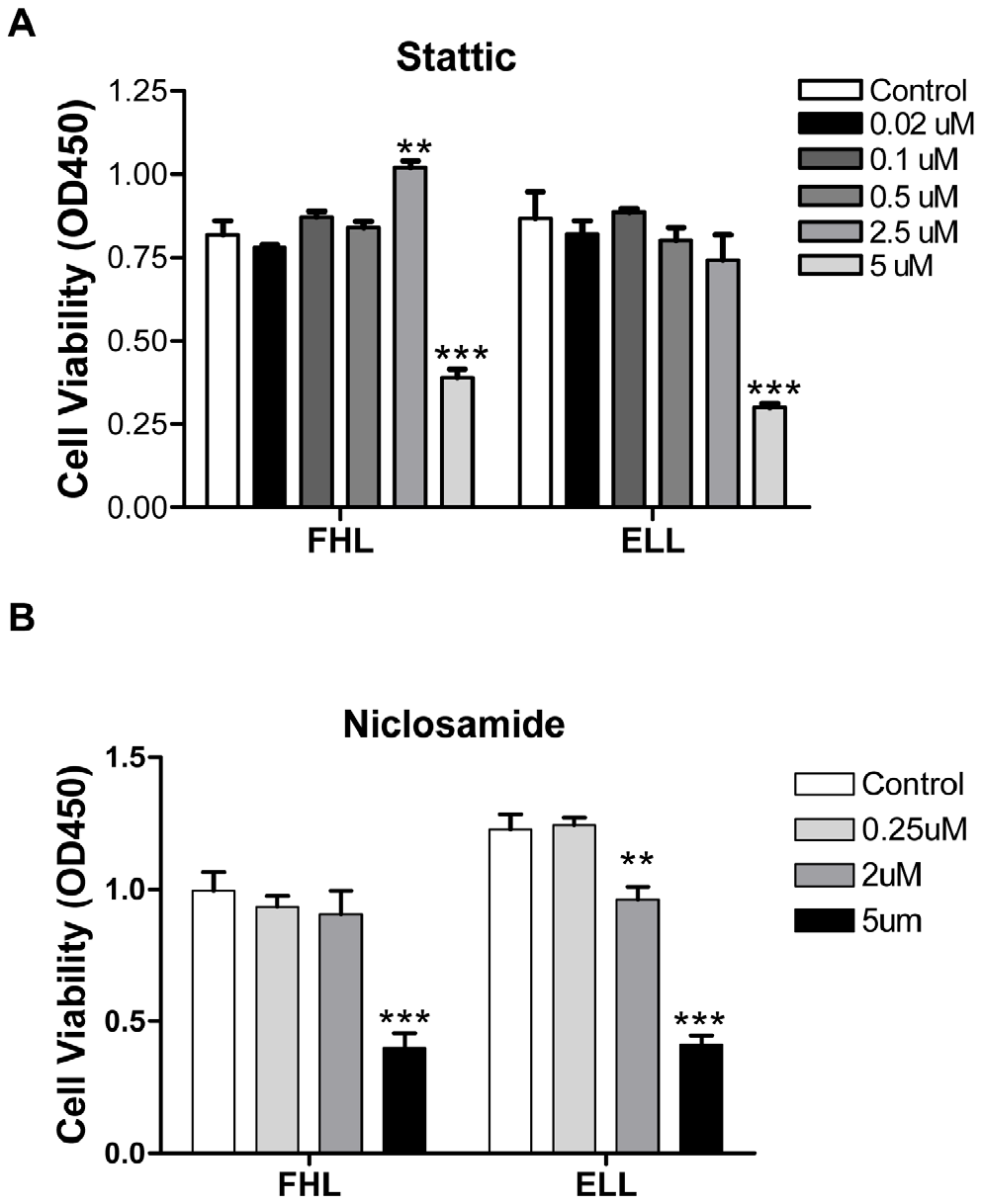
Stattic and Niclosamide exhibit dose-dependent effects on hNSCs. Cell viability is determined by WST-1 assay in FHL- or ELL-primed hNSCs treated with varying doses of Stattic and Niclosamide. (**A**) Low doses of Stattic (up to 2.5 µM) did not affect viability in either FHL or ELL conditions. Higher concentrations of Stattic, such as 5 µM, are toxic to cells. (**B**) Low doses of Niclosamide reduced the number of viable cells in ELL-primed group whereas higher concentrations of Niclosamide (5 µM) were toxic to both FHL- and ELL-primed cells. Data are presented as mean ± SEM (n = 6), ***p<0.0001; **p<0.01 by One-way ANOVA with Tukey post-hoc tests. hNSCs: human neural stem cells; ELL: EGF plus LIF and laminin; FHL: FGF2 plus heparin and laminin.

To identify the optimal dosage of Stattic and Niclosamide that inhibit STAT3 activity but do not exhibit any morphological or cytotoxic effects, we performed morphological analysis and WST-1 viability assays in the presence of these inhibitors under EGF or FGF2 priming conditions. As previously reported, FHL-primed hNSCs had uniform cell size, shape and retained the ability to migrate (Fig. S1A) On the other hand, ELL-primed cells had diverse shapes, long radiating projections and limited migrating capacities (Fig. S1B, control). Upon treatment with various doses of Stattic, ELL- and FHL-primed hNSCs exhibited morphological features similar to their corresponding control cells (compare Fig. S1C to Fig. S1A and Fig. S1D to Fig. S1B). However, higher doses of Stattic resulted in cytotoxicity, under both ELL and FHL priming conditions (Fig. S1E, S1F). The WST-1 assay indicated that concentrations up to 2.5 µM Stattic did not affect cell viability whereas treatment with higher concentrations of Stattic led to the loss of cell viability in both ELL- and FHL-primed hNSCs (Fig. 2A). Similarly, cells treated with lower concentrations of Niclosamide and primed with ELL or FHL exhibited expected morphological features, well comparable to their corresponding controls (Compare Fig. S2A to Fig. S2C and Fig. S2B to Fig. S2D). However, both ELL- and FHL-primed hNSCs treated with higher concentrations of Niclosamide (2 µM and 5 µM) resembled FHL-treated cells more than ELL-treated controls (Fig. S2E and S2F). In addition, the WST-1 cell viability assay indicated that cells treated with 2 µM Niclosamide showed moderate cytotoxicity only in ELL-primed cells while higher doses (5 µM) of Niclosamide had significantly decreased viability when primed with either growth factor (Fig. 2B). Based on WST-1 cell viability and morphological analysis, sub-lethal doses of Stattic (0.5 or 2.5 µM) and Niclosamide (0.25 µM) were chosen for further studies.

### STAT3 Inhibition Increased Motor Neuron Differentiation in hNSCs

**Figure 3:**
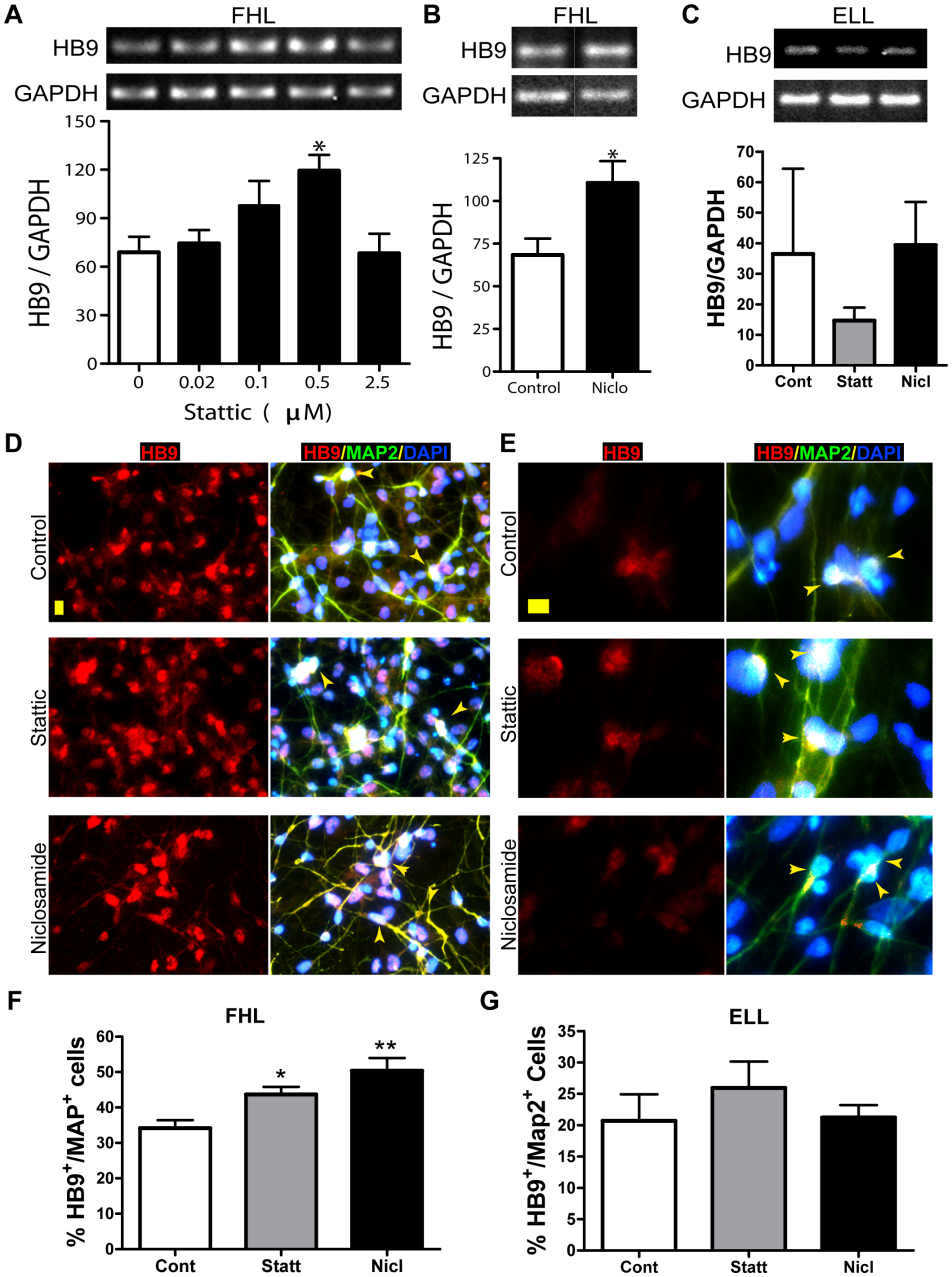
Increased motor neuron differentiation in hNSCs by STAT3 inhibition. (**A–C**) Semi-quantitative RT-PCR analyses of the expression level of HB9 mRNA, a marker for spinal motor neurons, after a 4-day priming. GAPDH mRNA expression serves as internal control. In general, FHL-primed hNSCs (**A,B**) express more Hb9 mRNA than the corresponding ELL-primed cells (**C**)**.** Hb9 mRNA levels are significantly increased in FHL-primed hNSCs treated with 0.5 µM Stattic (**A**) or 0.25 µM Niclosamide (**B**). In contrast, STAT3 inhibition does not alter HB9 mRNA expression in ELL-primed cells (**C**). Values are mean ± SEM (n = 3), *p<0.05, One-way ANOVA plus Tukey post- hoc tests (A and C) or Student’s *t*-test (B). (**D–G**) Immunofluorescent staining of Hb9/MAP2-labeled motor neurons in hNSCs primed and inhibitor-treated for 4 days and differentiated in B27 for 9 days. Hb9 immunoreactivity is in general higher in FHL-primed than ELL-primed cells. Representative epifluorescent microscopic images show increased Hb9 and MAP2 immunoreactivity in FHL-primed hNSCs after treatment with Stattic (0.5 µM) and Niclosamide (0.25 µM) (**D**). Inhibition of STAT3, on the other hand, does not affect HB9/MAP2 labeling in ELL-treated cells (**E**). Arrowheads point to cells that are co-labeled with Hb9 and MAP2, and contain a DAPI-stained nucleus with cytoplasm extended into neurites. Scale bar: 10 µM (**F–G**) Quantitative analyses show that 0.5 µM Stattic significantly increase the percentage of Hb9^+^/MAP2^+^ cells in FHL-primed cells. *p<0.05 by One-way ANOVA with Tukey post-hoc tests. hNSCs: human neural stem cells; ELL: EGF plus LIF and laminin; FHL: FGF2 plus heparin and laminin; Statt: Stattic; Nicl: Niclosamide.

To test if STAT3 inhibition affects motor neuron specification of hNSCs, we analyzed mRNA and protein expression levels of Hb9, a known marker of motor neurons. To compare Hb9 mRNA levels upon STAT3 inhibition, cells primed with either ELL or FHL were treated with various sub-lethal concentrations of either Stattic or Niclosamide for 24 hrs followed by RT-PCR analysis. We observed a consistently significant increase in Hb9 mRNA levels in FHL-primed cells with increasing concentrations of Stattic (Fig. 3A). Treatment with 0.5 µM Stattic resulted in roughly double the Hb9/GAPDH ratio compared to untreated FHL controls (Fig. 3A). However, further increase in Stattic concentrations did not increase Hb9 mRNA yield, indicating that this concentration of Stattic provides the level of STAT3 that results in optimal expression of Hb9 mRNA. Similarly, we observed a dose-dependent increase in Hb9 mRNA levels upon Niclosamide treatment and found that FHL-primed cells treated with 0.25 µM Niclosamide had significantly increased the Hb9/GAPDH ratio compared to FHL-primed untreated cells (Fig. 3B). On the other hand, treatment with Stattic (0.5uM) or Niclosamide (0.25 uM) in ELL-primed cells did not result in any significant increase in Hb9/GAPDH ratio (Fig. 3C). This indicates that precise control of STAT3 levels along with FGF2 leads to optimal Hb9 mRNA expression. Moreover, as reported previously, the ratio of Hb9/GAPDH in FHL-primed control cells is usually at least 2-fold higher than the ratio of Hb9/GAPDH in ELL-primed control cells (Fig. 3A–B Control vs Fig. 3C Control). To confirm that the increase in Hb9 mRNA levels in FHL-primed hNSC treated with optimal doses of Stattic or Niclosamide was accompanied by a corresponding increase in Hb9 protein levels, immunofluorescent analysis was performed. Human neural stem cells were subjected to either ELL- or FHL-priming followed by a nine-day differentiation period in B27 media. Subsequently, the cells were double immunolabeled with antibodies against Hb9, an early marker for motor neurons and MAP2 (microtubule-associated protein 2), which marks mature differentiated neurons. We observed that ∼35% of FHL-primed control cells expressed both Hb9 and MAP2 (Fig. 3D,F control). Upon inhibition of STAT3, it was increased to ∼45% in FHL-primed 0.5 µM Stattic-treated cells (Fig. 3D,F) and 50% in FHL-primed cells treated with 0.25 µM Niclosamide (Fig. 3D, F). However, ELL-primed cells did not exhibit a significant increase in motor neuron specification upon STAT3 inhibition using either Stattic or Niclosamide (Fig. 3E, G). As graphically represented in Fig. 3G, ∼20% of ELL-primed control cells co-localized for both Hb9 and MAP2. About 25% and 20% HB9^+^/MAP2^+^ cells were found in Stattic- and Niclosamide-treated groups, respectively. Our newly developed STAT3 inhibitor, HJC0149, with a much lower cellular toxicity and improved bioavailability [18], [19], also exhibited similar increases in the Hb9 transcript level as well as the number of Hb9^+^/MAP2^+^ motor neurons in the presence of FHL (Fig. S3). Thus, we conclude that inhibition of STAT3 increased the transcript levels of Hb9 as well as the number of differentiated motor neurons only in FHL-primed cells but not in ELL-primed cells.

### Stattic and Niclosamide do not Affect Tyrosine Phosphorylation of STAT3 but Perturb its Nuclear Translocation

**Figure 4:**
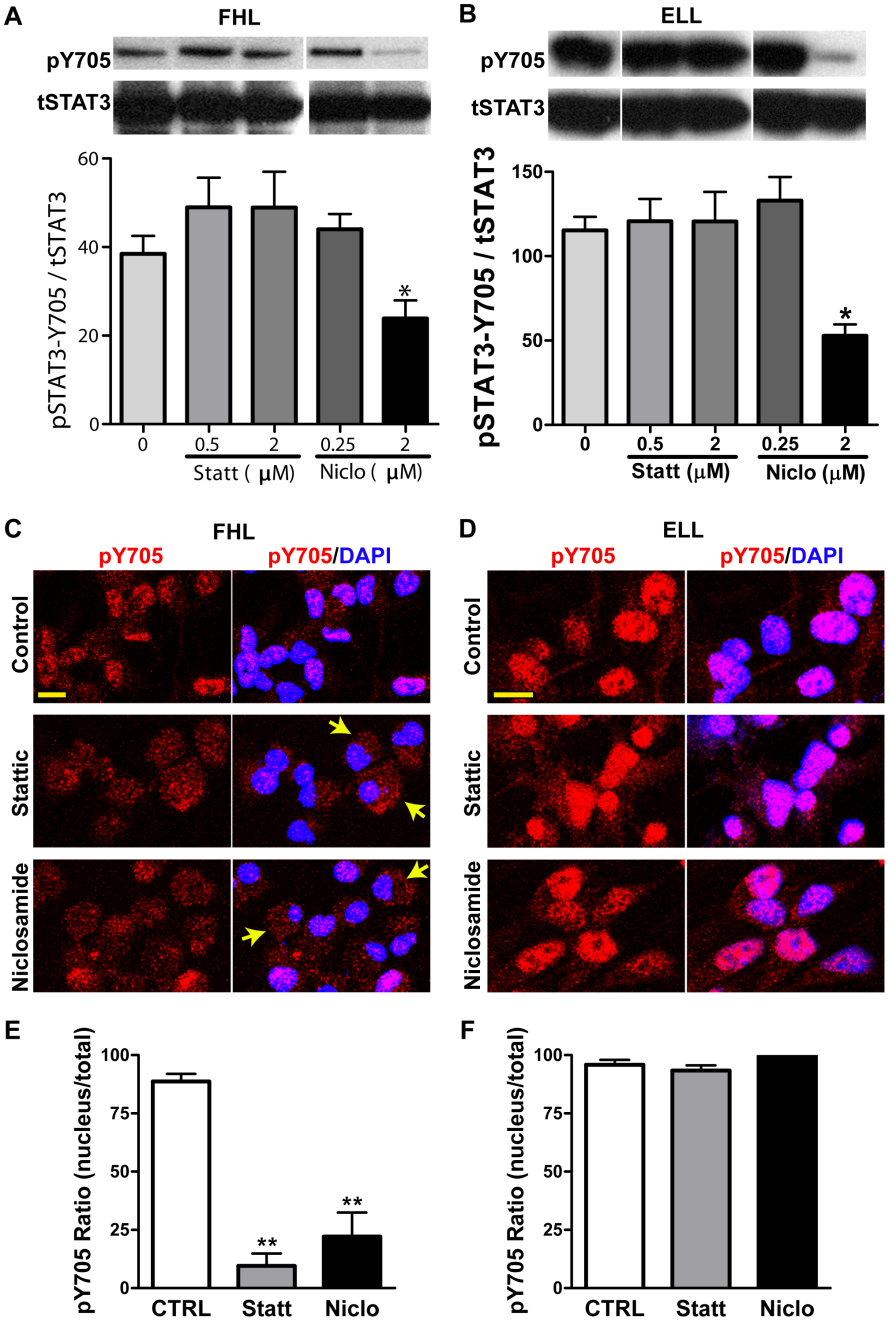
STAT3 phosphorylation in hNSCs treated with STAT3 inhibitors. (**A–B**): Western blot analyses of pSTAT3-Tyr705 (pY705) and total STAT3 (tSTAT3) in FHL- or ELL-primed hNSCs, with or without treatment of Stattic and Niclosamide for 24 hrs. GAPDH was used as loading control. Densitometric analysis was performed and normalized values of pSTAT3-Y705/tSTAT3 calculated from 3 independent experiments are graphically represented (Mean ± SEM). In general, FHL-primed hNSCs exhibit a much lower level of pY705 (**A**) than that in ELL-primed cells (**B**). STAT3 inhibitors, Stattic and Niclosamide at the doses showing no cytotoxicity, do not affect the phosphorylation of STAT3 at Y705 (pY705); whereas the cytotoxic dose of niclosamide resulted in a significant reduction of pY705 (n = 3, *p<0.05 by One-way ANOVA plus Tukey post-hoc tests). (**C–D**) Representative confocal images show the immunofluorescent staining of pY705 in hNSCs. Blue are nuclei counterstained with DAPI. pY705 (red) is localized mainly in nuclei of the FHL- or ELL-primed cells without STAT3 inhibitor treatment (Controls in **C** or **D**, respectively). Treatment with Stattic (0.5 µM) or Niclosamide (0.25 µM) decreases the nuclear translocation of activated STAT3 (pY705) in FHL- primed hNSCs (**C**), but not in ELL-primed cells (**D**). Scale bar, 10 µm. (**E–F**) Quantitative localization of pY705 STAT3 immunereactivity in the nucleus vs. in a whole cell. **p<0.01. One-way ANOVA with Dunnett post hoc tests. hNSCs: human neural stem cells; ELL: EGF plus LIF and laminin; FHL: FGF2 plus heparin and laminin; Statt: Stattic; Niclo: Niclosamide.

Tyrosine phosphorylation of STAT3 leads to its dimerization via phosphotyrosine interactions, which alters its conformation, allowing for target DNA binding and activation of downstream transcriptional targets. Thus, to identify the mechanism as to how Stattic and Niclosamide disrupt STAT3 pathway activity, we first hypothesized that these inhibitors may disrupt tyrosine phosphorylation of STAT3. To test that, we performed an immunoblot analysis of ELL- and FHL-primed hNSCs treated with various concentrations of Stattic and Niclosamide with an antibody specific to tyrosine 705-phosphorylated form of STAT3. As expected, we observed an overall increase in the levels of pSTAT3-Y705 in FHL-primed cells versus ELL-primed control cells (Fig. 4A,B. “0” panels). However, contrary to our expectations, there was no significant difference in the levels of pSTAT3-Y705 among various sub-lethal doses of Stattic or Niclosamide irrespective of whether they were primed with FHL or ELL (Fig. 4A,B). While a higher dose (2 µM) of Niclosamide resulted in decreased pSTAT3-Y705 levels, it was attributed to perturbed morphological changes (Fig. S2E,F) that indicate some unexplained underlying physiological changes and hence were not taken into consideration. The levels of pSTAT3-S727, total STAT3 and GAPDH (loading control) remained unchanged in ELL-primed, FHL-primed controls and corresponding drug treated cells (data not shown). Thus, Stattic and Niclosamide do not affect tyrosine phosphorylation of STAT3.

A recent study demonstrated that nuclear import of STAT3 occurs independent of tyrosine phosphorylation of STAT3 [21]. Therefore, we asked if nuclear translocation of tyrosine phosphorylated form of STAT3 was adversely affected in the presence of STAT3 inhibitors. If this was indeed the case, we expected to see decreased levels of nuclear tyrosine-phosphorylated STAT3 upon treatment with these STAT3 inhibitors. To test this, ELL- and FHL-primed hNSCs treated with 0.5 µM Stattic or 0.25 µM Niclosamide were immunostained with a phosphor-specific antibody against tyrosine-705 phosphorylated STAT3 and counter-stained for DAPI, a fluorescent dye that acts as a nuclear marker. We observed that in FHL-primed control cells, pTyr705-STAT3 was largely localized to the nucleus, although some of it was also observed in the cytoplasm (Fig. 4C). Interestingly, about 75% of Stattic-treated cells exhibited cytoplasmic localization of pSTAT3-Y705 and only about 25% had pSTAT3 in the nucleus (Fig. 4C,E). This block in nuclear transport was also observed in FHL-primed Niclosamide-treated cells (Fig. 4C). On the other hand, almost all of the ELL-primed control cells exhibited nuclear pSTAT3-Y705 (Fig. 4D) with the level of immunoreactivity much stronger than that in FHL-primed cell nuclei (Fig. 4C). Upon treatment with sub-lethal concentrations of Stattic or Niclosamide, ELL-primed cells exhibited no significant change in the amount of nuclear localized pSTAT3-Y705 when compared to ELL-primed control cells (Fig. 4D,F). Thus, Stattic and Niclosamide, block nuclear transport of pSTAT3-Y705, specifically in FHL-primed cells, but not in ELL-primed cells. This difference in subcellular localization correlates well with the increase in motor neuron specification only in STAT3 inhibitor-treated, FHL-primed cells. Taken together, it appears that through an unknown mechanism, increased levels of cytoplasmic tyrosine phosphorylated STAT3 may result in an increase in motor neurons.

### STAT3 Inhibition in hNSCs Decreased GFAP^+^ Astrocytes

**Figure 5:**
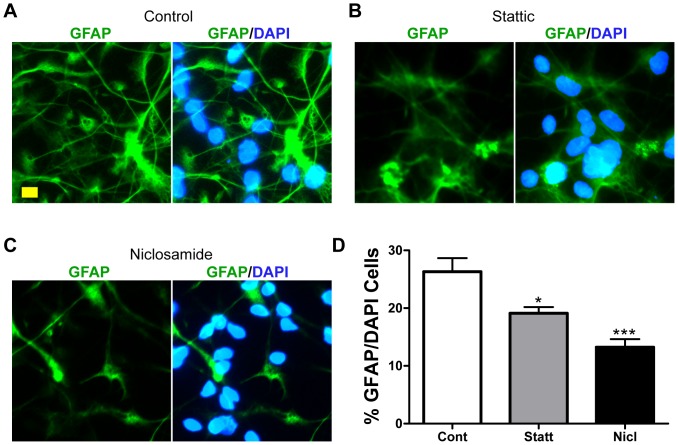
Astroglial differentiation in hNSCs treated with STAT3 inhibitors. Immunofluorescent staining is used to determine GFAP-labeled astrocytic differentiation in human neural stem cells (hNSCs) that were FHL-primed and inhibitor-treated for 4 days, and then differentiated in B27 for 9 days. (**A–C**) Representative epifluorescent microscopic images show GFAP immunoreactivity in FHL-primed hNSCs (**A**) or after treatment with Stattic (0.5 µM) (**B**) and Niclosamide (0.25 µM) (**C**). Scale bar, 10 µm. (**D**) Quantitative analyses show that 0.5 µM Stattic and 0.25 µM Niclosamide significantly decrease the percentage of GFAP^+^ cells. *p<0.05 and ***p<0.001 by One-way ANOVA with Tukey post-hoc tests.

Previous studies have shown that STAT3 binding to the glial fibrillary acidic protein (GFAP) promoter is essential for GFAP expression [22], [23]. We hypothesized that reduced nuclear levels of STAT3 in FHL-primed Stattic or Niclosamide hNSCs should reduce GFAP expression and hence, the number of GFAP^+^ astrocytes. To test that, we treated control, FHL- or ELL-primed hNSCs with Stattic or Nicloasmide. After a 9-day differentiation period, they were fixed and co-immunostained with antibodies against GFAP, an intermediate filament protein that marks mature astrocytes, and the nuclear marker, DAPI. It was observed that ∼30% of FHL-primed control cells were positive for GFAP (Fig. 5A, D). FHL-primed hNSCs treated with 0.5 µM Stattic (Fig. 5B, D) or 0.25 uM Niclosamide (Fig. 5C, D) exhibited significantly reduced ratio of GFAP^+^/DAPI (Fig. 5D). This clearly indicates that sub-lethal doses of Stattic and Niclosamide in the presence of FGF2, are sufficient to reduce GFAP expression and thereby, astroglial differentiation, most likely due to reduced nuclear pSTAT3-Y705.

## Discussion

The main findings of our study are that 1) hNSCs primed with FGF2 had reduced levels of phosphorylated/activated STAT3; 2) Treatment of FGF2-primed, but not EGF-primed, hNSCs with specific pharmacological inhibitors of STAT3 enhanced motor neuron fate specification at the expense of astroglial differentiation; 3) The STAT3 inhibitors, Stattic and Niclosamide, do not inactivate STAT3 signaling by affecting STAT3 expression or its phosphorylation, but by blocking nuclear transport of activated STAT3.

### FGF2 Reduced Levels of Phosphorylated STAT3

FGF2 is an essential neurogenic factor required for proliferation and differentiation of multipotent neural stem cells both during development and in the adult mouse brain [24], [25]. Moreover, it is known that in the adult central nervous system (CNS), FGF2 is expressed in the neurogenic niches (the subventricular zone of the lateral ventricles–SVZ; and the subgranular zone of the hippocampal dentate gyrus–SGZ) and has been implicated in the control of adult neurogenesis based on changes in proliferation and differentiation of adult neural stem and progenitor cells [25], [26]. The role of FGF2 in NSC propagation and proliferation both *in vivo* and *in vitro* is well established [26], [27]. Previous studies from our lab demonstrated that FGF2 and EGF have differential effects on motor neuron fate specification in human fetal neural stem cells, achieved by fine modulation of phosphatidylinositol 3-kinase (PI3K)/Akt/glycogen synthase kinase 3β (GSK3β) signaling, independently of MAPK and PLC-γ pathway [15], [20]. The current model to explain the differential effects of FGF2 and EGF on hNSCs is that FGF Receptor-induced Akt/GSK3β is lower than the corresponding EGF Receptor-induced Akt-GSK3β activation. This results in lowered phosphorylation of GSK3β at Ser9 and therefore, leads to increased transcription of Hb9, a crucial homeobox transcription factor crucial for the identity of postmitotic motor neurons, likely via Ngn2 phosphorylation/activation [20].

Interestingly, we found that priming hNSCs with FGF2 and heparin results in dramatic reduction in tyrosine phosphorylation of STAT3. It is known that STAT3 regulates the expression of GFAP, a key component of astrocytes. In early telencephalic neuroepithelial cells and postmitotic neurons, activation of GFAP expression is blocked by CpG methylation of its STAT3 binding sites, and demethylation of STAT3 binding sites leads to GFAP expression [22], [23]. FGF2 regulates ciliary neurotrophic factor (CTNF)-mediated induction of GFAP expression via STAT3 binding but does not affect CNTF-induced STAT1/3 tyrosine-705 phosphorylation [28]. Thus, the reduction in tyrosine-705 phosphorylation of STAT3 that we observed upon FGF2-priming of hNSCs occurs via CNTF-independent mechanisms.

### Blocking STAT3 Increases Motor Neuron Differentiation of FGF2-primed hNSCs

Our results demonstrate that optimal doses of pharmacological inhibitors of STAT3 in FGF2-primed hNSCs result in increased Hb9 mRNA expression and increased Hb9- and MAP2-positive mature neurons. At the same doses, Stattic and Niclosamide fail to promote motor neuron generation in EGF-primed hNSCs. This indicates that FGF2 together with STAT3 inhibition enhances motor neuron differentiation, whereas EGF blocks motor neuron differentiation, which further confirms our previous reports [15], [20]. Alternatively, since we observed that ELL-primed hNSCs have very high levels of tyrosine phosphorylated/activated STAT3 to begin with, STAT3 inhibitors may be required at much higher concentrations to reduce pSTAT3 levels sufficiently to allow for motor neuron differentiation. However, such high concentrations result in pleiotropic effects including altered morphology, inhibited migration and even cytotoxicity. Thus, it appears that fine tuning of the STAT3 signaling pathway along with reduced toxicity, enhanced bioactivity and medical availability is necessary for modulating the gliogenic environment and enhancing endogenous neural repair. In an effort to explore new STAT3 inhibitors with higher specificity and bioavailability toward potential clinical usages, we have developed a new class of STAT3 inhibitors [18], [19], [27]. One of the compounds, HJC0149, increased HB9 mRNA expression and HB9^+^/Map2^+^ neuronal differentiation, and also decreased GFAP^+^ astrogliogenesis of hNSCs with very little cytotoxicity (unpublished data).

Our data are consistent with previous reports in that STAT3 suppression or its conditional deletion in cultured neural stem cells promotes neurogenesis [11]. In those studies, Hb9 was not explored, and only the more generic MAP2 expression in differentiated neurons was assessed. Our study has thus demonstrated for the first time the role of the STAT3 pathway on Hb9 transcription and protein expression, which in vertebrates is specifically expressed in somatic motor neurons and is essential for distinguishing motor neuron/interneuron subtypes.

In previous studies, STAT3 was reduced either by inoculation of cultured rat embryonic brain neurospheres with adenovirus overexpressing a dominant negative form of STAT3 (Stat3F) [11] or by adenoviral infection of NSC prepared from STAT3-conditionally deleted mouse embryos with nuclear *Cre* recombinase [12]. In both cases, loss of STAT3 resulted in significant downregulation of Notch family members (Notch 1,2,3, hes5, Id3). In the developing CNS, Notch signaling preserves the progenitor pools and inhibits neurogenesis. Thus, it is plausible that increased Hb9 mRNA and protein expression that we observed upon STAT3 inhibition was a result of decreased Notch activity in human neural stem cells. However, the mechanism by which STAT3 activity could impact Notch signaling during neurogenesis remains to be determined.

In contrast to these studies, a recent finding suggests that STAT3 activation is needed for motor neuron differentiation during early development of the spinal cord [29]. This suggests that perhaps STAT3 performs different roles during neural stem cell maintenance and motor neuron differentiation. Alternatively, this provides further support for our hypothesis that maintaining precise spatial and temporal control *in vivo* and exact stoichiometric control of STAT3 under *in vitro* conditions is the key to obtaining maximum motor neuron differentiation. In addition, it is pertinent to note that the cellular environments during neurogenesis versus in injured/degenerating adult neurons are completely different and hence may not be directly comparable.

### Stattic and Niclosamide Do Not Affect Phosphorylation of STAT3 but Perturb its Nuclear Translocation

We observed that treatment of hNSCs with pharmacological inhibitors, Stattic and Niclosamide, did not affect STAT3 phosphorylation status at either Tyr-705 or Ser-727. Typically, phosphorylation of STAT3 is considered the hallmark of the activated STAT3 signaling pathway. Although much effort has been made in developing STAT3 inhibitors that block its phosphorylation, there are known STAT3 inhibitors that block STAT3 pathway activity by disrupting other molecular steps. For instance, pyrimethamine affects STAT3 transcriptional activation without affecting its tyrosine phosphorylation [30].

Recent studies demonstrate that STAT3 is different from other members of the STAT family in that while tyrosine phosphorylation is critical for its DNA binding ability, nuclear import of STAT3 occurs independent of its phosphorylation status [21]. Consistent with this, we observed that upon treatment with Stattic and Niclosamide in FGF2-primed cells, nuclear localization of pSTAT3-Y705 was significantly reduced with a concomitant increase in its levels in the cytoplasm. Interestingly, ELL-primed cells exhibit a much higher baseline level of pSTAT3-Y705 nuclear localization, which was not changed by treatments with similar concentrations of Stattic or Niclosamide. Thus, we conclude that it is most likely that Stattic or Niclosamide inhibits STAT3 activity by blocking nuclear import and, presumably, by decreasing nuclear accumulation of pSTAT3-Y705, which somehow results in increased motor neuron specification.

### STAT3 Inhibition Decreases GFAP^+^ Astrocytes

Our results indicate that inhibition of STAT3 using optimal doses of STAT3 inhibitors, Stattic or Niclosamide, decreased GFAP^+^ astrocyte populations in EGF- and FGF2-primed hNSCs. Activated STAT3 is an important cue for glial differentiation [12], [31], [32]. Various external cues like cytokines, CNTF, bone morphogenetic proteins, leukemia inhibitory factor, and Notch-Delta ligands activate the STAT3 pathway through JAK kinase [33], [34] ERK [35], mTOR (Bone Morphogenetic Proteins) [36] or directly through Notch [37], which regulates GFAP expression by epigenetic chromatin remodeling. Moreover, it has been demonstrated that mouse cortical progenitors stimulated with FGF2 exhibit increased GFAP expression and astrocyte formation; this is due to hypermethylation of lysine4, and thereby increased STAT3 binding to the GFAP promoter [22], [23], [28]. Thus, it is plausible that in our paradigm of FGF2 priming followed by STAT3 inhibition, there is an insufficient nuclear pool of pSTAT3 to bind to GFAP promoter sites (owing to blocked nuclear import), which results in decreased GFAP expression and thereby, astroglial differentiation.

In summary, our study provides insights into how neural stem cells can be manipulated *in vitro* to obtain more motor neurons. We show that a combination of precise level of STAT3 activity, obtained by STAT3 inhibition along with increased FGF2 levels result in an enhanced generation of motor neurons. Presumably, this study will have broader implications to stem cell therapies designed to ameliorate the negative effects of spinal cord injury and ALS by increasing motor neuron production in a more favorable environment.

## Supporting Information

Figure S1 **Dose-dependent morphological effect of Stattic on ELL- and FHL-primed hNSCs.** Representative phase contrast images of hNSCs following a 3-day priming in FHL (**A, C** and **E**) or ELL (**B, D** and **F**), with or without the treatment of a STAT3 inhibitor, Stattic. Low doses of Stattic (up to 2.5 µM) did not affect cell morphology. Scale bar, 20 µm.
(TIF)

Figure S2 **Dose-dependent morphological effect of Niclosamide on ELL- and FHL-primed hNSCs.** Representative phase contrast images of hNSCs after a 3-day priming in FHL (**A, C** and **E**) or ELL (**B, D** and **F**), with or without the treatment of a STAT3 inhibitor, Niclosamide. Low dose of Niclosamide (0.25 µM) did not affect cell morphology or cell viability. 2 µM Niclosamide not only blocked cell migration and inhibited process formation in both FHL and ELL conditions. Scale bar, 20 µm.
(TIF)

Figure S3 **Increased motor neuron differentiation in hNSCs by a novel STAT3 inhibitor, HJC0149.** (**A**) Chemical structure of HJC0149. (**B**) Semi-quantitative RT-PCR to determine the expression level of HB9 mRNA after 4-day priming. GAPDH used as an internal control. Hb9 mRNA levels are significantly increased in FHL-primed hNSCs treated with 0.5µM HJC0149 (HJC). Values are mean ± SEM (n = 3), *p<0.05, One-way ANOVA plus Bonferroni post-hoc tests. (**C**) Quantitative analyses show that 0.5µM HJC0149 significantly increase the %Hb9^+^/MAP2^+^ cells in FHL-primed cells by immunostaining. *p<0.05 compared to the control (CTRL), Student’s *t* test. (**D–E**) Representative epifluorescent microscopic images to show HB9/MAP2-labeled motor neurons in hNSCs primed alone (**D**) and primed plus inhibitor-treated (**E**) for 4 days and differentiated in B27 for 9 days. Scale bar, 20 µm. DAPI, nuclear counterstain; HB9, transcription factor and motor neuron marker; MAP2, microtubule associated protein 2.
(TIF)
