# Supplementary figures and images for: STAT3 Modulation to Enhance Motor Neuron Differentiation in Human Neural Stem Cells
Source: PLoS One. 2014 Jun 19;9(6):e100405. doi: 10.1371/journal.pone.0100405 (PMC4063761; doi:10.1371/journal.pone.0100405)

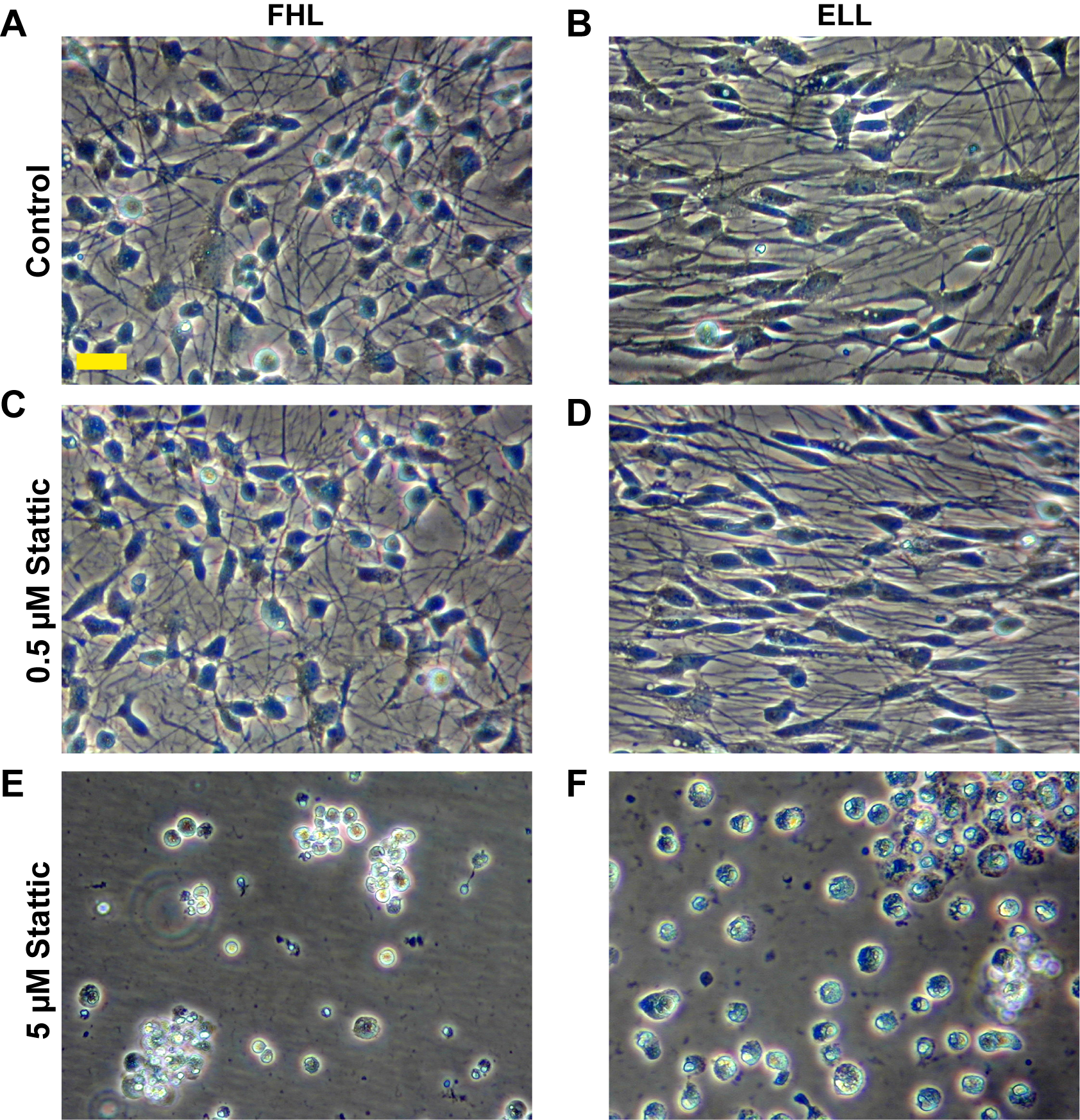

Supplement: Figure S1 — Dose-dependent morphological effect of Stattic on ELL- and FHL-primed hNSCs. Representative phase contrast images of hNSCs following a 3-day priming in FHL (A, C and E) or ELL (B, D and F), with or without the treatment of a STAT3 inhibitor, Stattic. Low doses of Stattic (up to 2.5 µM) did not affect cell morphology. Scale bar, 20 µm. (TIF) [file pone.0100405.s001.tif]

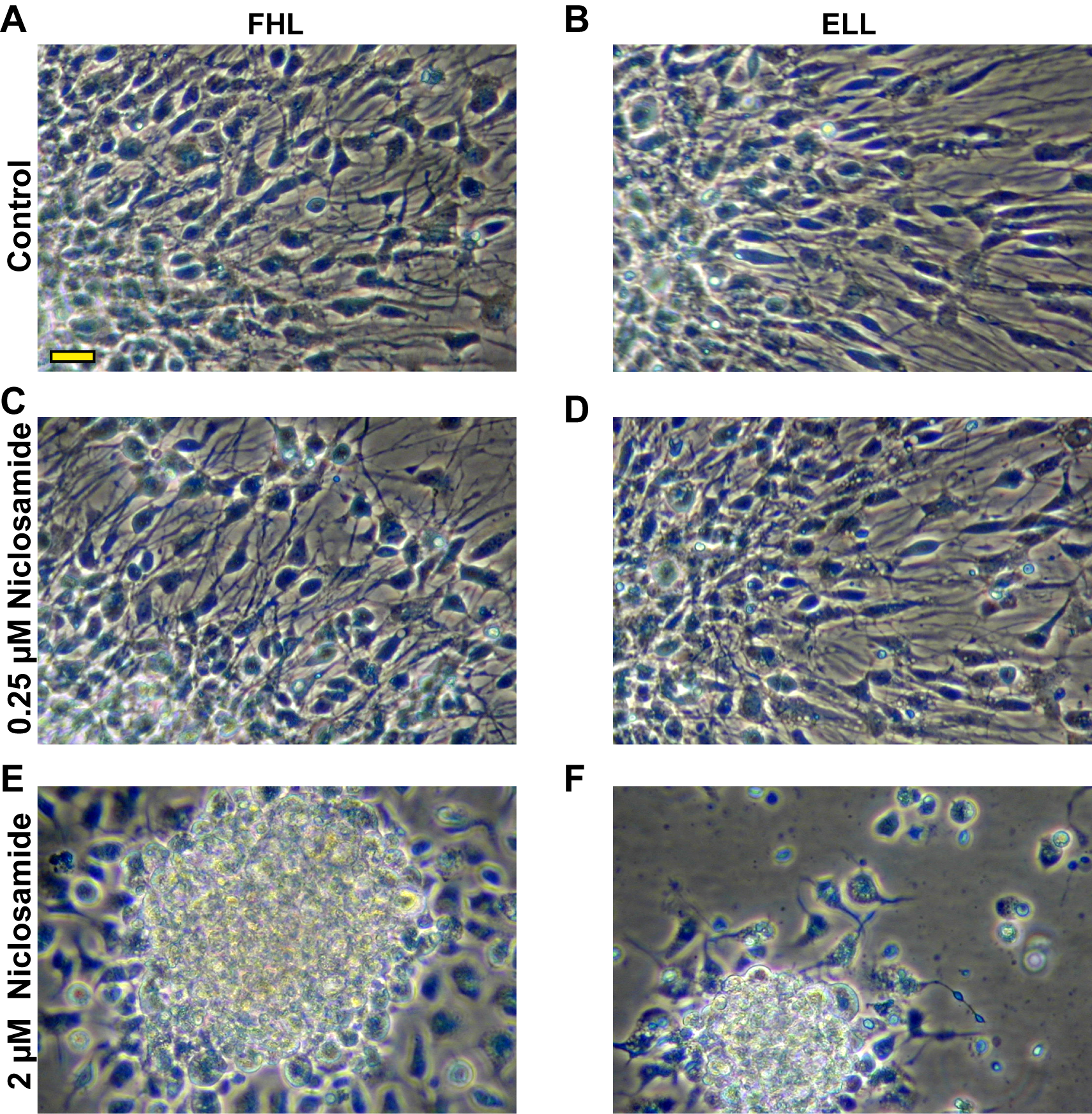

Supplement: Figure S2 — Dose-dependent morphological effect of Niclosamide on ELL- and FHL-primed hNSCs. Representative phase contrast images of hNSCs after a 3-day priming in FHL (A, C and E) or ELL (B, D and F), with or without the treatment of a STAT3 inhibitor, Niclosamide. Low dose of Niclosamide (0.25 µM) did not affect cell morphology or cell viability. 2 µM Niclosamide not only blocked cell migration and inhibited process formation in both FHL and ELL conditions. Scale bar, 20 µm. (TIF) [file pone.0100405.s002.tif]

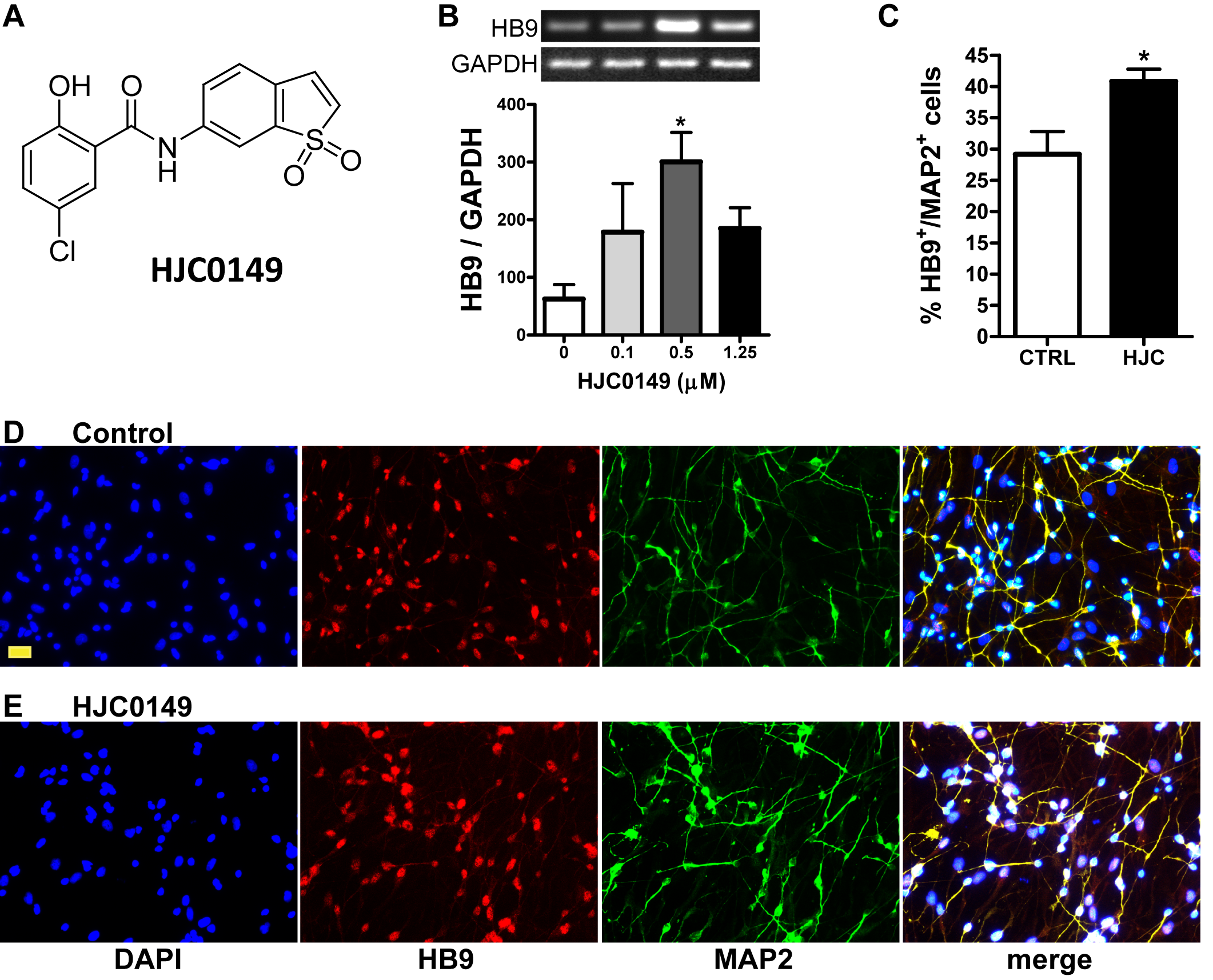

Supplement: Figure S3 — Increased motor neuron differentiation in hNSCs by a novel STAT3 inhibitor, HJC0149. (A) Chemical structure of HJC0149. (B) Semi-quantitative RT-PCR to determine the expression level of HB9 mRNA after 4-day priming. GAPDH used as an internal control. Hb9 mRNA levels are significantly increased in FHL-primed hNSCs treated with 0.5µM HJC0149 (HJC). Values are mean ± SEM (n = 3), *p<0.05, One-way ANOVA plus Bonferroni post-hoc tests. (C) Quantitative analyses show that 0.5µM HJC0149 significantly increase the %Hb9+/MAP2+ cells in FHL-primed cells by immunostaining. *p<0.05 compared to the control (CTRL), Student’s t test. (D–E) Representative epifluorescent microscopic images to show HB9/MAP2-labeled motor neurons in hNSCs primed alone (D) and primed plus inhibitor-treated (E) for 4 days and differentiated in B27 for 9 days. Scale bar, 20 µm. DAPI, nuclear counterstain; HB9, transcription factor and motor neuron marker; MAP2, microtubule associated protein 2. (TIF) [file pone.0100405.s003.tif]
